# Quality of family planning counseling and associated factors among reproductive age women who are current contraceptive users at Dessie town health facilities east Amhara, 2023

**DOI:** 10.1186/s12913-024-11833-z

**Published:** 2024-11-05

**Authors:** Nigusie Abebaw, Berihun Haile, Amare Workie, Wondwosen Mebratu, Molla Getie

**Affiliations:** 1 Department of Midwifery, College of Medicine and Health Sciences, Wollo University, Dessie, Ethiopia; 2 Dessie Comprehensive Specialized Hospital, Dessie, Ethiopia; 3 Department of Epidemiology and Biostatistics, College of Medicine and Health Sciences, School of Public Health, Wollo University, Dessie, Ethiopia; 4 Department of Medical laboratory science, College of medicine and Health sciences, Injibara University, Injibara, Ethiopia

**Keywords:** Quality of FP counseling, Dessie town health facilities

## Abstract

**Background:**

The role of counseling in Family Planning is to care a woman in navigating the process of choosing a contraceptive method that will allow her to fulfill her family planning goals and exercise her reproductive health rights. The effectiveness and appropriateness of family planning counselling play a crucial role in a client’s decision-making process regarding contraception. The decision for a client to use contraception with effectively and properly it should be ultimately achieved the quality of family planning counclling.

The aim of this research is to assess quality of family planning counseling and associated factors among reproductive age women at Dessie town health facilities, east Amhara, Ethiopia, and 2023.

**Methods:**

A facility based cross-sectional study was conducted from December 1, 2022- January 30, 2023. Study subjects were selected by using systematic random sampling method. Data was be collected by exit interview of the women and analyzed by SPSS version 26.  Bivariable and Multi variable logistic regression were executed to identify associated factors with quality of family planning counseling and the P- Value <0.05 on Multi variable analysis was considered as significantly associated with the dependent variable.

**Result:**

The proportion of women receiving good quality family planning counselling in this study was 36.5%.  Contraceptive source [AOR=2.03, 95%CI (1.09, 3.75)], Contraceptive currently used [AOR=0.43, 95%CI (0.26, 0.73)], separate room for family planning counseling [AOR=3.38, 95%CI (1.09, 3.75)] and availability of all methods [AOR=3.10, 95%CI (1.85, 5.21)]were significantly associated with quality of family planning counselling.

**Conclusion:**

The proportion of women obtaining good quality FP counseling in this study low. Type of contraceptives currently used, source of contraceptives, separate room for providing family planning counseling and availability of all methods of family planning in the facility are significantly associated with quality of family planning counseling. Therefore, all health profession could be given proper counseling to increase the quality of family planning.

**Supplementary Information:**

The online version contains supplementary material available at 10.1186/s12913-024-11833-z.

## Introduction

Quality has been defined at a clinical level, and involves offering technically competent, effective and safe care that pays to the client’s well-being [1]. According to the United States Institute of Medicine, “Quality of health care is the degree to which health services for individuals and populations increase the likelihood of desired health outcomes and are consistent with current professional knowledge” [1, 2].

Good quality family planning service helps individuals and couples to meet their reproductive health need safely and effectively [3]. Quality of care in family planning methods encompasses a wide range of problems including technical competence, choice of methods, information given to clients, interpersonal relationships and appropriate constellation of services [4]. Availability of resources including materials and skilled personnel affect the process of Family Planning service delivery which consequently predicts the service quality [5].Moreover, consumers of family planning have the right to be treated with privacy and dignity, receive information and the chosen contraceptive methods safely [2].

Globally, 225 million women who want to prevent pregnancy are not using real and safe contraceptive methods [4]. Also, the above efforts, the family planning service must consider clients’ satisfaction, which has not been considered much. Because accessibility of health service facilities will not necessarily guarantee the utilization of health services; utilization also depends on the client’s readiness to access the service and their satisfaction toward the service [6].

A study across 15 sub-Saharan countries showed that within one year of starting a family planning method, 7–27% of women ceased to use contraception for reasons related to the information received and confusion about side effects [7]. Therefore, imperative to provide high-quality counseling that fosters the provision of proper information about family planning and contraception to clients.

As of recent estimâtes, Dessie town has a population of approximately 535,000, with a significant proportion being of childbearing âge of 258,00 [8]. The town has experienced rapid population growth in recent years. Keep record of any Dessie Town community efforts or programs that have successfully enhance maternal health via family planning advice. This could involve collaborations with NGOs, health education campaigns, or community-based initiatives.

In order to ensure efficient contraceptive use and improve the state of reproductive health, family planning counseling is essential. Recent studies conducted in the location of East Amara’s Dessie Town demonstrates the crucial role of high-quality counseling for impacting patterns of contraceptive use. Prior studies have demonstrated variations in the caliber of family planning advice offered in different industries and medical institutions. Nevertheless, exact causes of this variation are not well-documented, especially when it comes to Dessie Town. Numerous research concentrate on broad evaluations of counseling quality rather than exploring the subtleties unique to a given area.

Nevertheless the contraceptive service in the country especially in Dessie town is still deficient and characterized by poor counselling quality for contraceptive services. Therefore the aim of this finding is to assess quality of family planning counselling and factor associated among reproductive age women at Dessie town health facilities, east Amhara, Ethiopia, and 2023.

## Methods and materials

### Study area and period

This study was conducted from December 1, 2022 to January 30, 2023 at selected public and private health facilities located in Dessie town, which is located at 401 Km far from Addis Ababa on the north east of Ethiopia. It sits at a latitude and longitude of 11°8′N 39°38′E, with an elevation between 2,470 and 2,550 m above sea level.

The total population of Dessie town is estimated to be 535,000 ,from those nearly half 258,00 are females [9]. Currently providing services given more than 10 million people including South Wollo, North Wollo, Waghimra and Oromiya specialized zones.

According to the report from Dessie town health department, there are 2 public hospitals, 4 private hospitals, 5 public health centers, 7 private specialized clinics and 2 nonprofit non-governmental organizations providing current modern contraceptive users of nearly 13,000 women in the study area.

### Study design

An institution based cross-sectional study was conducted to employ this research.

### Source population

All childbearing age women who are current users of modern contraceptive at Dessie town health facilities.

### Study population

All childbearing age women who visited the selected family planning clinic of Dessie town during the study period.

### Eligibility criteria

#### Inclusion criteria

Age between 15 and 49 years Women who visited the selected facility for obtaining family planning service and who are current users of one of the family planning methods.

#### Exclusion criterion

Women who are seriously ill and not voluntary to give response during data collection period.

### Sample size determination

For the first objective, sample size was calculated by single population proportion formula assuming a 95% confidence level.

$$\frac{\mathrm n=\;{\mathrm Z^2}_{\mathrm\alpha/2\;}\mathrm X\;\mathrm P\left(1-\mathrm P\right)}{\mathrm d^2}$$

Where: n = the required sample size,

Z = a standard score corresponding to 95% confidence level;

P = proportion of women who get quality family planning counseling, which was 33.4% from a previous study conducted in Ethiopia [10].

d = margin of error = 0.05.

$$\frac{\mathrm n=\;\left(1.96\right)^2\;\times\;0.334\left(0.666\right)=342}{\left(0.05\right)^2}$$

For the second objective, sample size was calculated by using epi info version 7.2.5 stat calc for cohort and cross-sectional studies by assuming 95% confidence level and 80% power by considering variables significantly associated with quality of Family planning counseling from previous studies (Table 1).

**Table 1:** Sample size calculation for the second objective of the study on quality of family planning counseling and associated factors among reproductive age women who are current contraceptive users at Dessie town health facilities, east Amhara, Ethiopia, 2023

Variable	Ratio of unexposed: exposed	% out come among exposed	OR	Sample size	reference
Method source	1:1	14.3%	0.58	170	(10)
Method type	1:1	25.4%	0.31	328	(10)
Residence	1:4	29.8%	1.51	288	(10)

Since the sample size calculated for the first objective is larger than sample size calculated for the second objective, the final sample size for the study was 342.

### Sampling technique and procedures

Systematic random sampling was applied to guarantee a representative sample of medical institutions and individuals from the healthcare system in Dessie Town. The initial step was to identify all healthcare facilities operating with in Dessie Town. This list was compiled from several sources, including public health record, local health département directories, and Professional associations.

Facilities off ring a range of services, including primary care, specialized care, and emergency services, were considered. This diversity was crucial for understanding the breadth of services provided across the town.

After establishing the list of eligible facilities, we employed systematic random sampling. The process involved:

The total number of women receiving Family Planning services in the 6 facilities of Dessie town over two months were 1050 and the study subjects proportionally allocated were 342, the sampling interval of kth value for this study was 1050/342 was 3.So the sample was collected every 3 interval up to completing of the total sample size .The first women was selected by simple random sampling method.

### Variables of the study

#### Dependent variable

- ➢Quality of family planning counseling.

#### Independent variables

- ➢ Socio-demographic factors: Age, marital status, birth history, residence and media exposure.
- ➢ Contraception-specific factor: method type.
  ➢ Service delivery point factors: Source of contraceptive (Non-Governmental Vs Public), having separate room for providing family planning services, availability of all methods.

### Operational definitions

Quality of family planning counseling: was assessed by using the six indicator questions that forms the basis of the Method Information Index (MII). quality of counseling in two categories levels based on the number of “yes” (coded 1) responses to the equally weighted questions that form the basis of the Method Information Index (MII) [10].

Poor counseling was defined as score less than or equal to the mean score (<=3) of the six indicator questions, and good counseling was defined as score above the mean score (>3) of the six indicator questions [10].

### Data collection tools and procedures

A structured questionnaire which includes questions that assess study variables used to prepared and adapted from different literatures and standard tools including socio- demographic factors, contraceptive factors and service delivery point factors [1, 2, 4, 11–13] .The Johns Hopkins School of Public Health Center for Communication Programs IEC Research Tools [14]. Data was collected by using face to face interview women who visit the selected facility of Family planning clinic by one trained health professional (BSc nurse) who don’t work at family planning clinic for each selected facility, and supervised by the principal investigator.

Knowledge related to Family planning counclling was assessed using nine “yes/no” questions with scale of reliability of (α) = 0.72. The composite score was dichotomized using the median as the cut-off value, where a score equal to or above the median was considered “adequate knowledge about family planning counclling, and a score below the median was considered inadequate knowledge.

### Data quality control

To assure quality, a structured validated questionnaire was developed in English after reviewing relevant literature, and translated to the local language (Amharic) and back translated to English language to check for its consistency. In addition, data collectors and supervisors were trained prior to data collection for one day regarding technique, ethics of data collection, and data collection process by the principal investigator. Pretest was done on 5% of reproductive age women who are current contraceptive users at Woldia town before the actual data collection. Data collectors, at the end of the data collection session, checked the questionnaires for completeness. In order to avoid data loss, all questionnaires were stored in locked cabinets throughout the study and accessed only by the principal investigator.

### Data processing and analysis

Data was coded and entered using Epi-data Version 4.6 software, then exported to SPSS version 26 for further statistical analysis. Both descriptive and analytic analyses were executed. Descriptive characteristics were described in terms of mean (standard deviation) and median (interquartile range) for continuous data and frequency distribution for categorical data. Frequency tables, graphs, and cross-tabulation were used to present the findings of the study. Both bivariable and Multi variable logistic regression models were used to identify factors associated with quality of family planning counseling and those variables having *P* value ≤ 0.25 during bivariable analysis were entered into the multivariable analysis. *P*- Value < 0.05 on Multi variable analysis were considered as statistically significant. Model fitness was checked by using Hosmer and Lemshow statistic. Both crude odds ratio (COR) and adjusted odds ratio (AOR) with 95% confidence interval (CI) were computed to show the strength of association and to identify the main factors associated with quality of family planning counseling.

### Ethical consideration

Ethical clearance letter was obtained from the ethical review committee of College of Medicine and Health Sciences, Wollo University. A formal letter was submitted to all selected health facilities to obtain permission with the reference of “CMHSRCS/121/23”.

Before data collection Written Consent obtained from the study participants during data collection period. Increased risk factors for unwanted pregnancies, STIs, and other related health problems may result from this. We can provide 15-year-olds the tools and information they need to safeguard their health and make informed choices by involving them in health care. For participants who were under 18 years, informed assent was obtained from their parents or safeguards during data collection period. Kept strictly confidential and names of participants were not included in the questioner.

## Results

### Socio demographic characteristics of respondents

Response were obtained from 342 reproductive age women receiving family planning services from Dessie town health facilities making the response rate of 100%. The mean age of women was 28.2 years with SD of ± 7 years and the minimum and maximum age of women included in the study were 15 years and 46 years respectively. Out of the total study participants, more than half 204(59.6%) of them are urban residents, 249(72.8%) are married (Table 2).

**Table 2:** Socio demographic characteristics of respondents, 2023

Variable	Categories	Frequency	Percent
Age of women (in years)	15 to 19 years	36	10.5
	20 to 24 years	89	26.0
	25 to 49 years	217	63.5
Residence	Urban	204	59.6
	Rural	138	40.4
Marital status	Single	60	17.5
	Married	249	72.8
	Divorced and widowed	33	9.6
Birth history	No birth	104	30.4
	1 to 2 births	137	40.1
	3 and above births	101	29.5
Educational status	No formal education	54	15.8
	Primary education	69	20.2
	Secondary education and above	219	64.0
Occupation of women	House wife	133	38.9
	Self employed	112	32.7
	Non-governmental employee	25	7.3
	Governmental employee	72	21.1

### Contraceptive method related characteristics of women

Out of the total 342 study participants, the majority 288(84.2%) of them knows at least one types contraceptive methods. More than half 194(56.7%) of the respondents used short acting family planning methods and among the methods of contraceptives, the most widely used method was injectables being used by 105(30.7%) of the respondents and the least widely used method was sterilization method by 9(2.6%).

### Service delivery point related factors

Out of the total study participants, more than half 184(54.4%) of them had received their current (most recent) family planning method from public facilities, two third 239(69.9%) of them received services from facilities that have separate room for providing family planning services including counseling with only nearly one third 133(38.9%) of the facilities providing family planning services had all methods of family planning (Table 3).

**Table 3:** Contraceptive method and service delivery point related characteristics of women, 2023

Variable	Categories	Frequency	Percent
Know about any contraceptive	Yes	288	84.2
	No	54	15.8
Current or most recent contraceptive method used	Pills	66	19.3
	Injectables	105	30.7
	Implants	69	20.2
	IUD	67	19.6
	Sterilization	9	2.6
	Emergency contraceptives	26	7.6
Source of contraceptive	Public facilities	186	54.4
	Private facilities	**156**	45.6
The facility has separate room for FP counseling	Yes	239	69.9
	No	**103**	30.1
All FP methods are available in the facility	Yes	133	38.9
	No	209	61.1
Method of FP currently used	Short acting	194	56.7
	Long acting	148	43.3

### Quality of family planning counseling

Quality of family planning counseling was assessed by using six indicators adopted from the standard method information index (MII) tool used to assess quality of family planning counseling and a family planning counseling with total cumulative score of above the mean (> 3) considered as good quality counseling and a mean score or a score below the mean was considered as poor-quality counseling.

**Fig. 1:**
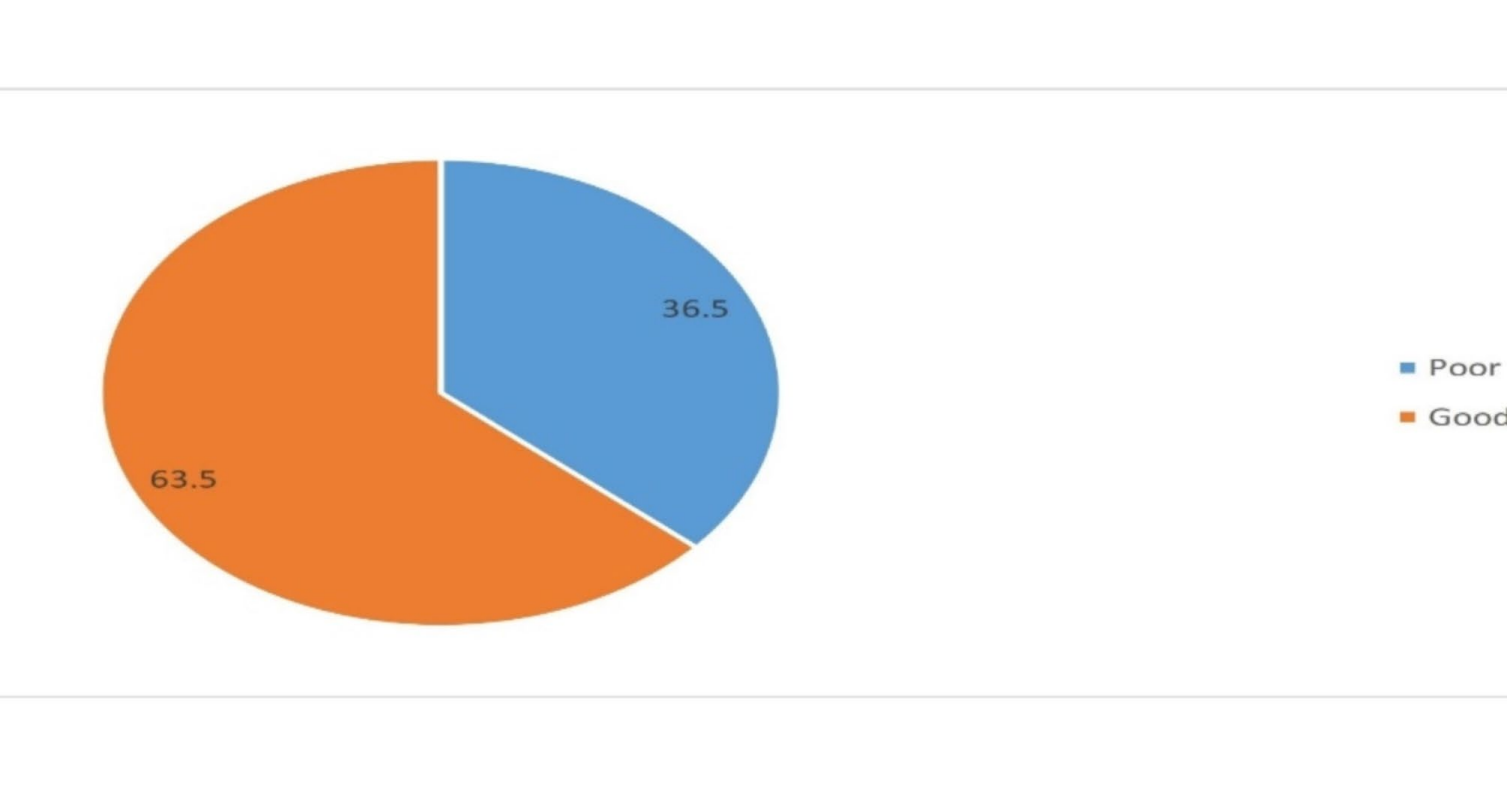
Quality of family planning counseling

Out of the six indicators, the most “yes” responses were obtained from indicator questions saying “Does your provider tell you when you come back?” and “Does your provider tell you about any method of family planning?” with a “yes” response of 201(58.8%) and 200(58.5%) respectively and the indicator with the least yes response was the question saying “Do you choose a method that suits your needs?” with a “yes” response of 123(36%). And finally, out of the total 342 women receiving family planning services, 125 (36.5%) of women surveyed reported receiving good quality family planning counseling (Fig. 1 and Table 4).

**Table 4:** Quality of family planning counseling, 2023

Variables/quality indicators	Categories	Frequency	Percentage
Does your provider tell you about any method of family planning?	Yes	200	58.5
	No	142	41.5
were you told by the family planning provider about methods other than the ‘most recent/current method’ that you could use?	Yes	157	45.9
	No	185	54.1
Do you choose a method that suits your needs?	Yes	123	36.0
	No	219	64.0
were you told by the provider about side effects or problems you might have with a method to delay or avoid getting pregnant?	Yes	161	47.1
	No	181	52.9
Were you told what to do if you experienced side effects or problems?	Yes	153	44.7
	No	189	55.3
Does your provider tell you when you come back?	Yes	201	58.8
	No	141	41.2

### Factors associated with quality of family planning counseling

Bivariable logistic regression was performed to select candidate variables for multivariable logistic regression analysis. Results of bivariable logistic regression showed that residence, knowledge about any contraceptive, educational status of women, type of contraceptives currently used, source of contraceptives, receiving FP services from facilities having separate room for providing family planning counseling and availability of all methods of family planning in the facility were candidates for multivariable logistic regression analysis with *P*-value < = 0.25.

multivariable logistic regression analysis result showed that, type of contraceptives currently used, source of contraceptives, receiving family planning services from facilities having separate room for providing family planning counseling and availability of all methods of family planning in the facility were significantly associated with quality of family planning counseling.

Mothers who received family planning services from public facilities are 2 times more likely to receive good quality family planning counseling compared to women who received family planning services from private facilities [AOR = 2.03, 95%CI (1.09, 3.75)].

Those women who are currently using short acting family planning methods had 57% decreased odds of good quality of family planning counseling compared to women who are currently using long acting family planning methods while keeping all other variables in the model constant [AOR = 0.43, 95%CI (0.26, 0.73)].

Those women who received family planning services from facilities having separate room for family planning counseling are 3.38 times more likely to receive good quality contraceptive counseling compared to women who received family planning services from facilities having no separate room for family planning counseling [AOR = 3.38, 95%CI (1.09, 3.75)].

Women who received family planning services from facilities all methods of family planning are available are nearly 3 times more likely to receive good quality contraceptive counseling compared to women who received from facilities all methods of family planning are not available [AOR = 3.10, 95%CI (1.85, 5.21)] (Table 5).

**Table 5:** Factors associated with quality of family planning counseling among women who are current contraceptive users at Dessie town health facilities, east Amhara, Ethiopia, 2023

Variable	Categories	Quality		COR (95%CI)	AOR (95%CI)	*P*-value
		Good	Poor			
Residence	Urban	98	106	3.80(2.30,6.28)	1.27(0.57,2.84)	0.563
	Rural	27	111	1	1	
know any contraceptive	Yes	110	178	1.61(0.85,3.05)	0.90(0.41,1.94)	0.781
	No	15	39	1	1	
Source of contraceptive	Public facility	89	97	3.06(1.91,4.90)	2.03(1.09,3.75)	0.025*
	Private facility	36	120	1	1	
Separate room for FP	Yes	112	127	6.10(3.24,11.52)	3.38(1.45,7.86)	0.005**
	No	13	90	1	1	
All methods available	Yes	70	63	3.11(1.97,4.92)	3.10(1.85,5.21)	0.000***
	No	55	154	1	1	
Type of contraceptive	Short acting	59	135	0.54(0.35,0.85)	0.43(0.26,0.73)	0.001***
	Long acting	66	82	1	1	
Educational status of women	No formal education	17	37	1	1	
	Primary education	19	50	0.83(0.38,1.80)	0.53(0.22,1.28)	0.157
	Secondary education and above	89	130	1.49(0.79,2.81)	1.35(0.66,2.78)	0.408

Significant *with *P*-value < 0.05 ** with *P*-value < 0.01 ***with *P*-value < 0.005

## Discussion

This study showed that the overall proportion of women who received good quality family planning counseling study was 36.5%. this finding is consistent with studies conducted in 25 developing countries [15] and another study conducted in Ethiopia [10]. But this finding is lower than studies conducted in Pakistan(73.4% ) [12], Asela town, Ethiopia (62.8%) [16] ,and studies conducted in Burkina Faso (63.4%) [17] .The possible reason for this finding studies mentioned above was due to difference in study setting and sampling technique and the other possible justification for the differences might be due to the socio demographic factors of each studies. Study done in Pakistan was used to only The Method Information Index (MII) during data collection period, but this study was used to both The Method Information Index (MII) and questioner and interview tools which was adapted from different literatures .studies done Burkina Faso was different socio demographic factors than the current study .women who are living in In Burkina Faso were good awareness about quality family planning counseling than the current study.

Those factors which are significantly associated with quality of family planning counseling in the study were type of contraceptives currently used, source of contraceptives, receiving FP services from facilities having separate room for providing family planning counseling and availability of all methods of family planning in the facility.

Using short acting family planning methods decreased odds of good quality of family planning counseling by more than 50% compared to currently using long acting family planning methods. This finding is consistent with two studies conducted in Ethiopia [10, 11]. Availability of all methods and receiving family planning services from facilities where all methods of family planning are available significantly increased the odds of good quality family planning counseling by three folds compared to women who had received their last (most recent) family planning services from facilities where all methods of family planning are not available. This finding is in line with WHO guidance and recommendations on the association of quality counseling and method availability [18], and the possible justification might be due to the fact that family planning counseling includes a series of steps which finally empowers the women to have informed choice.

Women who had received their current (most recent) family planning services from facilities having separate room for family planning counseling had three times increased odds of good quality family planning counseling compared to women who had received their current (most recent) family planning services from facilities having separate room for family planning counseling. This finding is in line with WHO guidance and recommendations on the association of quality counseling and method availability [18], and the possible justification might be due to the fact that privacy is the core element in family planning counseling which helps to create a conducive environment for sharing information .

Women who had received their current (most recent) family planning services from public facilities had two times increased odds of good quality family planning counseling compared to women who had received their current (most recent) family planning services from private facilities. This finding is in line with studies conducted in 25 developing countries [15] and Ethiopia [10].

### Limitation of this study

This research was done by institutional based cross-sectional study design, it is better to by community based cross sectional study design to address the whole community about quality of family planning counseling .There was also interviewers’ own prejudices or characteristics may unintentionally sway respondents’ responses. Interviewers may unintentionally guide interviewees toward particular replies, or their responses may be interpreted as passing judgment during data collection period. In addressing these potential biases and limitations, it is crucial to implement rigorous methodological strategies to enhance the reliability and validity of family planning research. By acknowledging these challenges and taking proactive steps to mitigate their impact, researchers can improve the accuracy of their findings and contribute valuable insights into family planning practices and policies.

## Conclusion

The proportion of women obtaining good quality FP counseling in this study was low compared with studies conducted in different countries.

Type of contraceptives currently used, source of contraceptives, receiving FP services from facilities having separate room for providing family planning counselling and availability of all methods of family planning in the facility are significantly associated with quality of family planning counselling. Therefore, all concerned body such as Government, health professionals, Researchers, Non-Governmental organizations should give special emphasis about family planning counselling method to increases the quality of services.

## Supplementary Information

Supplementary Material 1.

## Data Availability

The datasets supporting the conclusions of this article are included in the article.
