# Supplementary material for: Quality of family planning counseling and associated factors among reproductive age women who are current contraceptive users at Dessie town health facilities east Amhara, 2023
Source: BMC Health Serv Res. 2024 Nov 5;24:1345. doi: 10.1186/s12913-024-11833-z (PMC11536775; doi:10.1186/s12913-024-11833-z)
Supplement: Supplementary file 1 — Supplementary Material 1. [file 12913_2024_11833_MOESM1_ESM.docx]

# **ANNEXES**

**ANNEX 1. INFORMATION SHEET AND INFORMED CONSENT**

Dear respondent:

Hello, my name is ______________. Today I am here to collect data to assess the quality of family planning counseling among women who are current contraceptive users at Dessie town health facilities. I am asking you for a few minutes of your time to participate in this study. The information I interviewed and you give will be very useful in the realization of this study, and it will be kept confidential. your Name and Address will not be recorded. You have the right not to answer for any questions which might be inconvenient for you. However, your information is very important for the study. And we would like to confirm you that all your data are confidential and used for research purpose only.

Do you have any questions about this study?

Primary Investigator: Berihun Hailu Tell no: +251915199176

Thank you.

**CONSENT FORM (English version)**

The purpose of this study has clearly been explained to me. I understand what the study is about and what will be asked of me and I agree to participate. I understand that the records of this study will be kept confidential and will not include any records that can be used to identify me. I understand that taking part in this study is completely voluntary and that I can skip any questions which makes me feel uncomfortable for answering.

I have read or listened to the above information, and have received answers to all questions that I have asked. I give my consent to take part in this study.

Signature/thumb print of the mother: ____________________

Hospital Registration Number: _____________________

Signature of witness: _______________________

Date of interview (E.C.): ____/____/____

Name of the interviewer: __________________________

Signature of the interviewer: _______________________

Name of the supervisor: __________________________

Signature of the supervisor: _______________________

**ANNEX 2: ENGLISH VERSION QUESTIONNAIRE**

Good morning/afternoon? I am healthcare professional. I am collecting data regarding the quality of family planning counseling among women who are current contraceptive users at Dessie town health facilities, using structured questionnaires. It will take 15 to 20 minutes. Your honest response is very important to produce quality data in the organization there by to plan appropriate measures that could be taken.

Thank you for your participation.

ID of respondent _________________

MRN ____________________

| Part I: female client related factors | | |
| --- | --- | --- |
| 101 | Age of mother (years) | ___________ |
| 102 | Residence | 1. Urban 2. Rural |
| 103 | Marital status | 1. Married 2. Formerly in union 3. Never married |
| 104 | Educational status | 1. No formal education 2. Primary education 3. Secondary education and above |
| 105 | Occupation | 1. House wife 2. Self employed 3. Private employed 4. Government employee |
| 106 | Birth history | 1. No birth 2. 1 to 2 births 3. 3 and above births |
| **Part II: service delivery point and method related factors** | | |
| 201 | Do you know about contraception? | 1. Yes 2. No |
| 202 | If yes for Q 202, which type of contraception you know? | 1. Pills 2. Injectables 3. IUD 4. Implants 5. Sterilization 6. Emergency contraceptives 7. Others (specify)______ |
| 203 | What is the current/most recent contraceptive you use? | 1. Pills 2. Injectables 3. IUD 4. Implants 5. Sterilization 6. Emergency contraceptives |
| 204 | Source of contraception | 1. Private facility 2. Public facility |
| 205 | Does the facility have separate room for providing FP counseling service? | 1. Yes 2. No |
| 206 | All modern contraceptives available in the facility? | 1. Yes 2. No |
| **Part III: questions to assess quality of family planning counseling** | | |
| 301 | Does your provider tell you about any method of family planning? | 1. Yes 2. No |
| 302 | At that time (of using most recent method), were you told by the family planning provider about methods of family planning other than the ‘most recent/current method’ that you could use? | 1. Yes 2. No |
| 303 | Do you choose a method that suits your needs? | 1. Yes 2. No |
| 304 | When you obtained your ‘most recent/current method’, were you told by the provider about side effects or problems you might have with a method to delay or avoid getting pregnant? | 1. Yes 2. No |
| 305 | Were you told what to do if you experienced side effects or problems? | 1. Yes 2. No |
| 306 | Does your provider tell you when you come back? | 1. Yes 2. No |
